# Leptomeningeal metastatic cells adopt two phenotypic states

**DOI:** 10.1002/cnr2.1236

**Published:** 2020-01-29

**Authors:** Jan Remsik, Yudan Chi, Xinran Tong, Ugur Sener, Camille Derderian, Abigail Park, Fadi Saadeh, Tejus Bale, Adrienne Boire

**Affiliations:** ^1^ Human Oncology and Pathogenesis Program Memorial Sloan Kettering Cancer Center New York City New York USA; ^2^ Brain Tumor Center Memorial Sloan Kettering Cancer Center New York City New York USA; ^3^ Department of Neurology Memorial Sloan Kettering Cancer Center New York City New York USA; ^4^ Department of Pathology Memorial Sloan Kettering Cancer Center New York City New York USA

**Keywords:** cancer plasticity, cerebrospinal fluid, electron transport chain, leptomeningeal metastasis, metabolic adaptation

## Abstract

**Background:**

Leptomeningeal metastasis (LM), or spread of cancer cells into the cerebrospinal fluid (CSF), is characterized by a rapid onset of debilitating neurological symptoms and markedly bleak prognosis. The lack of reproducible in vitro and in vivo models has prevented the development of novel, LM‐specific therapies. Although LM allows for longitudinal sampling of floating cancer cells with a spinal tap, attempts to culture patient‐derived leptomeningeal cancer cells have not been successful.

**Aim:**

We, therefore, employ leptomeningeal derivatives of human breast and lung cancer cell lines that reproduce both floating and adherent phenotypes of human LM in vivo and in vitro.

**Methods and Results:**

We introduce a trypsin/EDTA‐based fractionation method to reliably separate the two cell subsets and demonstrate that in vitro cultured floating cells have decreased proliferation rate, lower ATP content, and are enriched in distinct metabolic signatures. Long‐term fractionation and transcriptomic analysis suggest high degree plasticity between the two phenotypes in vitro. Floating cells colonize mouse leptomeninges more rapidly and associate with shortened survival. In addition, patients harboring LM diagnosed with CSF disease alone succumbed to the disease earlier than patients with adherent (MRI positive) disease.

**Conclusion:**

Together, these data support mechanistic evidence of a metabolic adaptation that allows cancer cells to thrive in their natural environment but leads to death in vitro.

**Abbreviations**
AD: adherent cell fraction
ATP: adenosine triphosphate
BLI: noninvasive bioluminescence imaging
CSF: cerebrospinal fluid
CTC: circulating tumor cells
DE genes: differentially expressed genes
EDTA: ethylenediaminetetraacetic acid
FDR: false discovery rate
FL: floating cell fraction
LM: leptomeningeal metastasis
MRI: magnetic resonance imaging
PBS: phosphate‐buffered saline

## 1 INTRODUCTION

**FIGURE 1:**
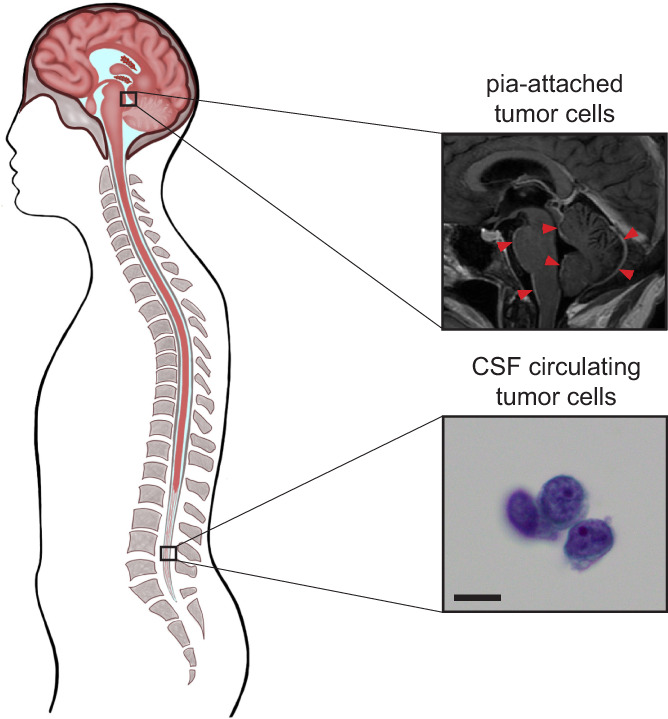
The two distinct cancer cell phenotypes of human leptomeningeal metastastasis. A, Human leptomeningeal metastasis from solid cancer primary. White plaques of leptomeningeal metastasis (LM) around pons, brainstem, and cerebellum (red arrows), as visualized by gadolinium‐enhanced magnetic resonance imaging (MRI), and Giemsa‐stained cytospin of cerebrospinal fluid (CSF) showing the cluster of cancer cells (bottom, scale bar = 5 μm)

Leptomeningeal metastasis (LM), or spread of cancer cells into the spinal fluid, is a fatal neurological complication of cancer characterized by unusually poor prognosis. Occurring in approximately 5% to 8% of patients with solid tumors and 5% to 15% of those with hematological malignancies, the prevalence of LM is increasing.^1^ Cancer cells may access the space through a variety of means, including passage through Bateson plexus via the venous circulation or choroid plexus via arterial circulation, direct invasion of spinal and cranial nerves, or spread from brain parenchyma through direct penetration of the glia limitans.^2^, ^3^, ^4^, ^5^ Once within this space, disseminated cancer cells can either attach to the meninges, as evidenced by linear or nodular enhancement on magnetic resonance imaging (MRI), or float freely within the cerebrospinal fluid (CSF), as seen on cytological examination and demonstrated by the presence of circulating tumor cells (CTCs) in spinal tap (Figure 1).^6^ Contemporary analyses of LM are limited to floating cancer cells since cancer cells adherent to meninges are retrieved only in rare autopsies. The extent to which floating cancer cells collected from the spinal fluid accurately represent the entire LM population (both floating and adherent) remains an open question. Paradoxically, although LM allows for feasible and longitudinal sampling by a spinal tap, a unique feature of this site of metastasis, attempts to culture patient‐derived leptomeningeal cancer cells have not met much success, suggesting that the floating cells may be adapted to this space.

In order to reproducibly study the dynamics of these adherent and floating LM subpopulations, we have employed leptomeningeal derivatives^7^ of human breast and lung cancer cell lines that reproduce both phenotypes of human LM in vivo and in vitro. To reliably separate the two cell subsets, we introduce a trypsin/EDTA‐based fractionation method. In vitro, we find that floating cells display a decreased proliferation rate, lower ATP content, and are enriched in distinct metabolic signatures when compared with their adherent counterparts. Long‐term fractionation and transcriptomic analysis suggest high degree plasticity between the two phenotypes in vitro. Floating cells colonize mouse leptomeninges more rapidly and associate with shortened survival. Remarkably, this finding is mirrored in a retrospective patient dataset: Patients with exclusively cytology (+) disease and MRI (−) disease succumbed more rapidly to LM than their counterparts with cytology (−), MRI (+), or cytology (+), MRI (+) disease. Together, these results support a model whereby cancer cells in LM exist in a plastic equilibrium between adherent and floating states; the floating phenotype represents the lethal variant of these cells.

## 2 MATERIALS AND METHODS

### 2.1 Human studies

CSF in excess of that required for clinical decision making was collected from patients undergoing lumbar puncture. Air‐dried cytospin CSF preparations were fixed in 4% PFA 5 minutes at room temperature, rinsed in PBS, and stained with Giemsa. Clinical data were obtained under MSKCC Institutional Review Board‐approved protocol 13‐039 “Gene expression patterns in Leptomeningeal Metastasis.” Clinical information, including tumor tissue diagnosis, coulter counter CSF counts, time to LM diagnosis, etc, was abstracted from the medical record and de‐identified. The presence of leptomeningeal disease in gadolinium‐enhanced MRI brain was evaluated based on hyperintense signal in the leptomeningeal space present on T1 postcontrast sequences and absent on T1 precontrast and susceptibility‐weighted sequences. Patients with incomplete pathological annotation (date and result of primary diagnosis, cytology/CTC count and brain and spine MRI, and date of death) and patients with intracranial metastases other than LM were excluded from further analyses. All patients provided informed consent.

### 2.2 Animal studies

Cell fractions of MDA‐MB‐231 (MDA‐231) LeptoM and PC9 LeptoM cells were prepared as described above. “Mixed” population of floating and adherent cells (mixed in 1:1 ratio prior to injection) was used as a control. The growth rate of unfractionated cells was characterized previously.^7^ All animal experiments were performed in accordance with protocol #18‐001‐02 approved by the MSKCC Institutional Animal Care and Use Committee. Athymic nude mice (Envigo) were housed in maximum barrier facility, with individually ventilated cages, up to five animals per cage with sterilized food and water and in 12 to 12‐hour light‐dark cycle. Mice were used at 5 to 8 weeks of age. MDA‐231 LeptoM cells were hosted in female mice and PC9 LeptoM cells in both female and male mice, in approximately 1:1 ratio. Cells were introduced intracisternally, using the procedure described previously.^7^ Briefly, deeply anesthetized mice were positioned prone over a 15‐mL conical tube to place cervical spine in flexion. The occiput was palpated, and a Hamilton syringe fitted with a 31G beveled (cutting) needle was introduced between the occiput and C1 at an angle. The needle was advanced 4 mm before introducing 10 uL of sterile PBS solution containing 2000 cells. The procedure was tolerated well with a success rate > 95%. The growth of cancer cells was monitored weekly, using bioluminescence imaging. D‐luciferin solution in PBS was delivered retro‐orbitally (potassium salt, GoldBio). Quantification of tumor burden by BLI was performed using an IVIS Spectrum‐CT (Caliper Life Sciences) and analyzed using Living Image software (v. 4.3.1, Perkin Elmer). Morbidity was monitored daily, and the development of neurological symptoms was used as a clinical end point in the survival studies.

### 2.3 Cell culture and cell fractionation

Parental and leptomeningeal (LeptoM) derivatives of PC9, MDA‐231, and HCC1954 cell lines were generated and characterized previously.^7^, ^8^, ^9^ Cells were routinely tested for mycoplasma contamination and assessed morphologically. Cell lines were derived in and obtained from the laboratory of Dr Joan Massague (MSKCC). PC9 is a model of EGFR^mut^/TP53^mut^ human lung cancer. MDA‐231 is a model of human triple‐negative (ER^−^PR^−^HER2^−^) breast cancer. HCC1954 is a model of human HER2/ERBB2‐driven (ER^−^PR^−^HER2^Amp^) breast cancer.

To feasibly purify and quantify fractions of cells that morphologically resemble floating or adherent phenotypes, we introduced an in vitro fractionation protocol using trypsin/EDTA. Similar strategies were reported and successfully reproduced previously.^10^ Briefly, 1.5 × 10^6^ cells were seeded into 100‐mm plate in 10‐mL complete culture medium. After 2 days, the plate was gently washed with sterile PBS and carefully incubated with trypsin/EDTA solution for either 3 minutes (for PC9 and HCC1954 cell lines) or 30 seconds at 37°C (MDA‐231 cell line). The trypsinated fraction was collected and assigned as “floating” (FL). The plate was again gently washed with PBS and incubated with trypsin/EDTA solution for another 5 to 7 minutes (for PC9 and HCC1954 cell lines) or 2 to 3 minutes (MDA‐231 cell line), and the resulting cell fraction was designated as “adherent” (AD). This procedure was successfully replicated by four investigators independently (J.R., X.T., C.D., and A.P.). In each experiment, the number of viable and dead cells was quantified using acridine orange/propidium iodide staining (Nexcelom ViaStain, #CS2‐0106). A fraction of floating cells was calculated as the total number of viable floating cells divided by the sum of viable floating and viable adherent cells, multiplied by 100, using the formula below:

$$\%\mathrm{FL}=\frac{\text{total}\;\mathrm{FL}}{\text{total}\;\left(\mathrm{FL}+\mathrm{AD}\right)}\;x\;100.$$

### 2.4 Proliferation and cytotoxicity assays

Unfractionated and fractionated cells were seeded into 96‐well plate, 1000 cells per well. Proliferation rate was measured on day 0, immediately after seeding (day 0 values served as normalization factor) and on days 4 and 9 after seeding for adherent (Corning, #3160) and nonadherent conditions (Corning, #3474), respectively, using CellTiter‐Glo Luminescent Cell Viability Assay as recommended (Promega, #G7572), and analyzed on GloMax (Promega). ATP per cell content was derived from day 0 measurements, employing ATP standard curve. ADDA‐5 HCl (Complex IV inhibitor) was acquired from Sigma‐Millipore (SML1940), reconstituted in DMSO and stored as recommended. Corresponding amount of DMSO was used as vehicle in all inhibitor studies. The testing range of all drugs was estimated based on two independent pilot experiments, followed by at least two independent experiments with optimized range. Half maximal inhibitory concentration values (IC_50_) were calculated in Prism (v7, GraphPad) using four‐parameter logistic regression.

### 2.5 Immunohistochemistry

Dissected brains were fixed in 10% neutral buffered formalin overnight and mounted into paraffin blocks; 5‐μm sections were stained using routine histology techniques. Antibodies used for immunostaining were chicken anti‐GFP (Aves, #1020), anti‐MTND5 (Abcam, #ab92624), anti‐MTCO1 (Abcam, #ab14705), and anti‐MTCO2 (Abcam, #ab79393). Secondary antibodies were conjugated with horseradish peroxidase, and DAB EqV Peroxidase was used as a substrate (all Vector Laboratories). Slides were scanned with MiraxScan (Zeiss), and images were analyzed using CaseViewer (v2.2, 3DHistech) and Fiji/ImageJ (v1.51j, NIH).

### 2.6 Transcriptomic analysis

RNA from fractionated PC9 LeptoM cells was extracted using RNeasy Mini Kit (Qiagen, #74136). After RiboGreen quantification and quality control by Agilent BioAnalyzer, 500 ng of total RNA underwent polyA selection and TruSeq library preparation according to instructions provided by Illumina (TruSeq Stranded mRNA LT Kit, #RS‐122‐2102), with 8 cycles of PCR. Samples were barcoded and run on a HiSeq 4000 in a 50/50 bp paired‐end run, using the HiSeq 3000/4000 SBS Kit (Illumina). An average of 49 million paired reads was generated per sample. Reads from generated FASTQ files were quality checked and mapped to the mouse reference genome (hg19) using STAR2.5.0.a. The expression count matrix of uniquely mapped reads was computed with HTseq v0.5.3. Raw counts were processed, batch effect was removed, and differentially expressed (DE) genes were analyzed with the EdgeR pipeline in RStudio (v1.0.143) with implemented R (v3.6.0) (Table S1). Normalized counts were extracted using DESeq2 package (v.1.24.0). Pathway analysis was performed with PANTHER Classification System (v14.0), using list of the DE genes with *P* < .01. Several DE genes were further validated independently by two investigators (J.R. and X.T.) using qPCR (Figure S3).

### 2.7 Messenger RNA detection

RNA was isolated as described above. To prepare cDNA, 2 μg of isolated RNA was transcribed using High‐Capacity cDNA Reverse Transcription Kit (Applied Biosystems, #4368814). Gene expression was determined using TaqMan Fast Advanced Master Mix (Applied Biosystems, #44‐449‐63) and annotated TaqMan gene expression assays (all Applied Biosystems): human MT‐ND5 (Hs02596878_g1), MT‐ATP6 (Hs02596862_g1), MT‐CO1 (Hs02596864_g1), and MT‐CO3 (Hs02596866_g1); and B2M (Hs00187842_m1) served as housekeeping genes.

### 2.8 Quantification and statistical analysis

Statistical analysis and figure plotting were performed in RStudio with implemented R (v. 3.5.1, CRAN) or Prism (v.7, GraphPad). The number of replicates for each experiment is annotated in figure legends. Reported values are averages ± standard error of the mean (SEM) or standard deviation (SD), as appropriate. Statistical analysis was performed by Student's *t* test, unless specified otherwise in corresponding figure legend. For pathway analysis of transcriptomic dataset, multiple hypothesis testing were adjusted using the Benjamini and Hochberg false discovery rate (FDR) method. Figures were assembled in Adobe InDesign CC2019.

## 3 RESULTS

### 3.1 Xenograft models reproduce floating and adherent phenotypes of human LM

**FIGURE 2:**
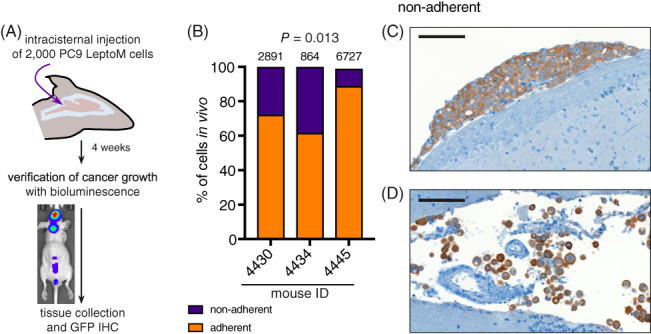
Mouse xenograft models of LM reproduce both phenotypes of human disease in vivo. A, Experimental schema. PC9 LeptoM cells expressing the TGL reporter were injected into the cisterna magna of athymic animals, and the tumor burden was monitored using noninvasive bioluminescence in vivo imaging weekly. The tissue was collected 4 weeks after injection and processed for immunohistochemistry. B, GFP‐expressing cancer cells in stained slides were counted using ImageJ and classified as “adherent” based on their tight adherence to pia mater or “nonadherent” based on their loose appearance. Portions of adherent and nonadherent cells were determined in four coronal brain sections in three animals, total number of counted cells per animal is shown above corresponding bars. C, Representative image shows adhered PC9 LeptoM cells (top) and freely floating cells and cellular clusters in vivo (bottom, scale bar = 200 μm)

**FIGURE 3:**
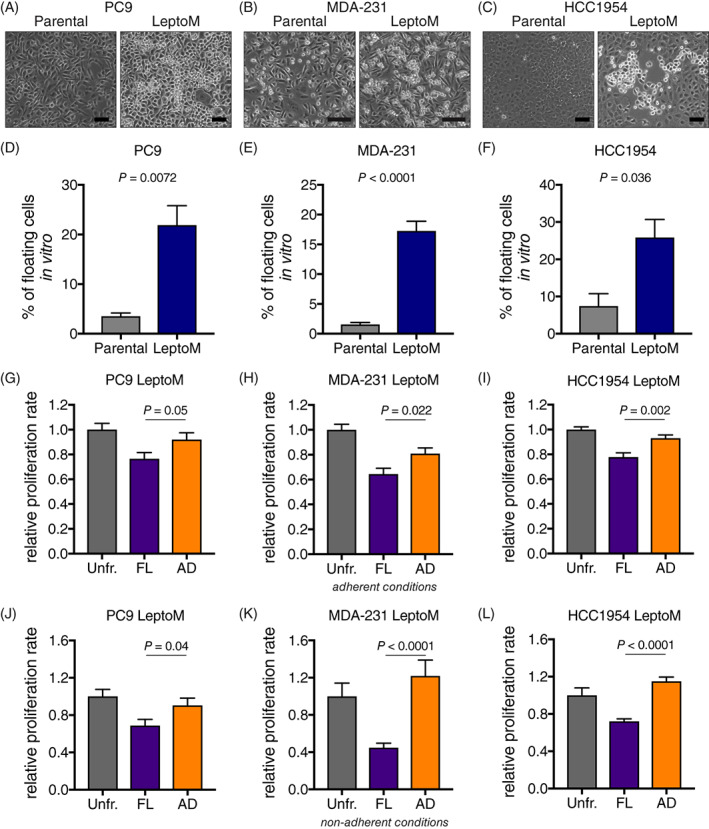
Floating leptomeningeal cancer cells have decreased proliferation *rate* in vitro. A‐C, Phase contrast images show in vitro morphology of parental and LeptoM PC9 (A), MDA‐231 (B), and HCC1954 cells (C; scale bar = 200 μm). D‐F, Plots show the portion of floating cells in cell culture of parental and LeptoM PC9 (D), MDA‐231 (E), and HCC1954 cells (F). Results are from at least three independent experiments, and data represent mean ± SEM. Please refer to Figure S1A for trypsin fractionation overview. G‐I, Plots show the relative proliferation rate of fractionated and unfractionated LeptoM PC9 (G), MDA‐231 (H), and HCC1954 cells (I), 4 days after seeding to adherent conditions (standard tissue culture plates). Results are from at least three independent experiments and data represent mean ± SEM. J‐L, Plots show the relative proliferation rate of fractionated and unfractionated LeptoM PC9 (J), MDA‐231 (K), and HCC1954 cells (L), 9 days after seeding to nonadherent conditions (ultralow attachment plates). Results are from four independent experiments and data represent mean ± SEM

**FIGURE 4:**
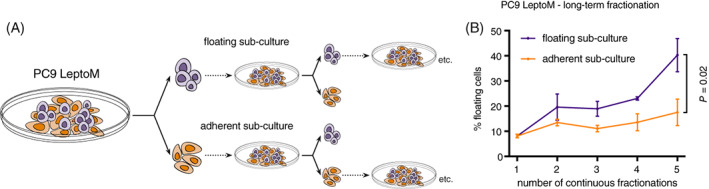
Continuous fractionation enriches for floating leptomeningeal cancer cells in vitro. A, Schema shows experimental strategy used for continuous fractionation. PC9 LeptoM cells were fractionated, and depicted fractions were seeded back to the cell culture. Subcultures were then again fractionated before reaching the confluency, and depicted fractions were seeded back to the cell culture. B, Plot shows the percentage of floating PC9 LeptoM cells in floating (violet) and adherent (orange) cell subcultures over the long‐term fractionation experiment, as described in (A). Results are from four independent experiments, and data represent mean ± SD

Patients suspected of harboring LM are generally evaluated by gadolinium‐enhanced MRI of brain and spine and CSF sampling, followed by cytological and/or flow cytometric evaluation of cancer cells (Figure 1). These complementary diagnostic approaches confirm the presence of two distinct cancer cell phenotypes in human LM. The cancer cells freely floating in the CSF and sampled with lumbar puncture grow in an anchorage‐independent manner. In contrast, cancer cells visualized on postcontrast MRI grow in sheets adherent to the pia mater. The relationship, function, and degree of heterogeneity and plasticity of these coexisting cancer populations remain unknown. To functionally dissect these two distinct cancer cell subtypes, we took advantage of unique leptomeningeal (LeptoM) derivatives of human lung and breast cancer cell lines.^7^ When injected intracisternally, PC9 LeptoM cells fully colonize the cranial cavity of immunodeficient mice within 4 weeks (Figure 2A). Morphological evaluation of cancer cells growing in the mouse CSF confirms the presence of both adherent (74.3%) (Figure 2B,C), and rounded, nonadhered cancer cells (25.4%) growing in pseudo‐clusters (Figure 2B,D). This phenomenon was present both in vivo and also in in vitro cell culture conditions (Figure 3A‐C). To reproducibly separate these fractions, we optimized a trypsin/EDTA‐based fractionation protocol (Figure S1A,B); for details, see Section 2. All tested LeptoM models were enriched in floating cells when compared with their parental counterparts (Figure 3D‐F). Floating and adherent cells collected from all tested cell lines showed similar viability values, as determined with acridine orange‐propidium iodide image analysis (Figure S1C‐E). Moreover, the floating fraction from all models demonstrated a decreased proliferation rate, under both adherent (Figure 3G‐I) and nonadherent conditions (Figure 3J‐L). Long‐term fractionation of both cell subsets derived from PC9 cell line (Figure 4A) led to the enrichment of floating cells in floating subculture, while fraction of the adherent subculture remained stable over time (Figure 4B).

### 3.2 The transcriptome of floating LM cells reveals distinct metabolic adaptations

**FIGURE 5:**
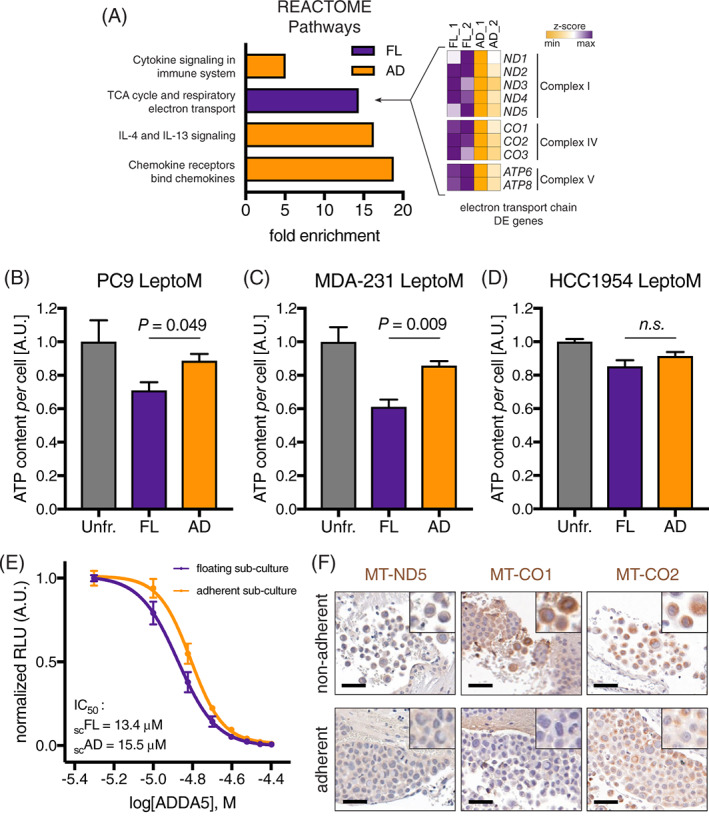
Transcriptomic analysis of floating leptomeningeal cancer cells reveals distinct metabolic adaptation. A, Plot shows significantly enriched REACTOME pathways in fractionated floating (FL) and adherent (AD) PC9 LeptoM cells (signature *P* < .01 and FDR < 0.01). Two independent samples per cell subset were sequenced. Differentially expressed genes related to the electron transport chain are plotted in the heatmap. B‐D, Plots show the per cell ATP content of fractionated and unfractionated LeptoM PC9 (B), MDA‐231 (C), and HCC1954 cells (D). Results are from at least three independent experiments, and data represent mean ± SEM. E, Plot shows the dose response of floating and adherent PC9 LeptoM subcultures to Complex IV poison ADDA5. Results are from three independent experiments, and data represent mean ± SEM. F, Representative images show expression of component of electron transport chain ND5, CO1 and CO2 in vivo, in adherent and nonadherent PC9 LeptoM cells (scale bar = 100 μm). Please also refer to Figure S3

To reveal the functional differences between floating and adherent cells, we performed transcriptomic analysis of fractionated PC9 LeptoM cells. The floating and adherent in vitro cultured PC9 LeptoM cells were strikingly similar based on DE genes (n = 184; *P* < .01); (Table S1), confirming high degree of plasticity and turnover between different phenotypes in in vitro conditions as seen after long‐term fractionation. Pathway analysis of DE genes revealed enrichment for genes involved in aerobic respiration and Krebs cycle in floating cells, confirmed by qPCR (Figure S3). In contrast, biological processes related to chemokine and cytokine signaling were enriched in adherent cells (Figure 5A). Consistent with this, we find that the floating population of PC9 LeptoM and MDA‐231 LeptoM cells contained less ATP (Figure 5B‐D). Moreover, the floating subpopulation of PC9 LeptoM cells displayed increased sensitivity to the electron transport chain poison ADDA5 (Figure 5E‐F).

### 3.3 Floating LM cancer cells exhibit an aggressive phenotype

**FIGURE 6:**
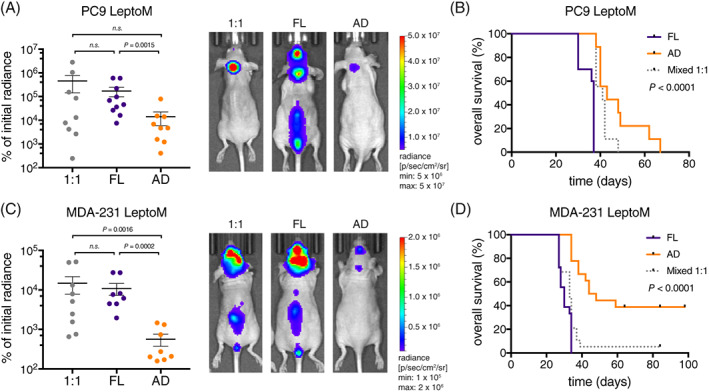
Floating leptomeningeal cancer cells exhibit more aggressive phenotype in vivo. A, Tumor growth and representative BLI images of mice injected with fractionated floating (FL), adherent (AD), and mixed (1:1) PC9 LeptoM cells 4 weeks after injection, n = 9 to 10 per group. Results are from two independent experiments. Data represent mean ± SEM, Mann‐Whitney *U* test. Please also refer to Figure S3A,B. B, Kaplan‐Meier survival curve of mice groups shown in (A). Log‐rank test. C, Tumor growth and representative BLI images of mice injected with fractionated floating (FL), adherent (AD), and mixed (1:1) MDA‐231 LeptoM cells 3 weeks after injection, n = 8‐9 per group. Results are from three independent experiments. Data represent mean ± SEM, Mann‐Whitney *U* test. Please also refer to Figure S3A,C. D, Kaplan‐Meier survival curve of mice groups shown in (D). Log‐rank test

To assess the phenotypic difference between the floating and adherent LeptoM cells in vivo, we injected equal numbers of floating, adherent, and mixed cells into the cisterna magna of immunodeficient animals (Figure S4A). In both PC9 lung (Figure 6A, Figure S4B) and MDA‐231 breast cancer LeptoM models (Figure 6C, Figure S4C), floating cells expanded in the cranial cavity more rapidly. Notably, the aggressiveness of floating cell in vivo was further demonstrated by accompanied by brisk development of neurological symptoms and decreased survival compared with their adherent or admixed counterparts (Figure 5B,D).

**FIGURE 7:**
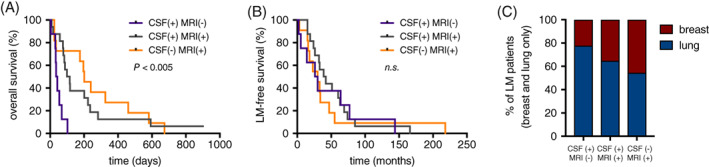
Floating leptomeningeal cancer cells represent lethal phenotype in lung and breast cancer patients. A, Kaplan‐Meier survival of lung and breast cancer patients initially diagnosed with CSF cytology alone (CSF +, MRI −, n = 8), MRI alone (CSF −, MRI +, n = 11), or both CSF and MRI (CSF +, MRI +, n = 16). B, Leptomeningeal metastasis‐free survival of patients shown in Figure 7A. C, Primary tumor type of patients shown in Figure 7A

Reasoning that a strong in vivo phenotype in mouse models may reflect human disease, we abstracted clinical information from the charts of 35 patients newly diagnosed with LM from breast cancer (n = 10), lung cancer (n = 23), or both (n = 2) (Table **S** **2**). Restricting our analysis to patients with complete LM staging at LM diagnosis, (MRI brain and spine +/− gadolinium and CSF with cytology), we generated three groups of patients: those with LM diagnosed by CSF cytology alone (CSF +, MRI −), those with LM diagnosed by MRI alone (CSF −, MRI +), and those with LM diagnosed by both indicators (CSF +, MRI +). Remarkably, patients diagnosed with CSF‐only disease demonstrated substantially diminished survival after LM diagnosis (Figure 7A). The onset of LM after primary diagnosis did not affect the site of LM (Figure 7B). However, lung cancer was more likely to present with CSF‐only disease (Figure 7C).

## 4 DISCUSSION

Metastatic colonization of distant body sites is an extremely inefficient biological process with fatal consequences.^11^ The hostile environment of secondary sites provides substantial selective pressure, requiring significant transcriptomic and epigenetic adaptations in cancer cells, mirrored by plastic changes in their phenotype.^12^ This phenomenon is particularly obvious in the case of LM, when typically adherent, tissue‐bound cancer cells spread and become nonadherent within the nutritionally sparse cerebrospinal fluid.^13^ Clinical observations and autopsy studies report that leptomeningeal cancer cells exist in two contrast phenotypes—freely floating in the CSF and adhering to leptomeninges and growing in plaques.^14^

The success in in vitro expansion of patient‐derived LM models is limited; attempts to reproduce these findings have gone unreported.^15^ The lack of in vivo and in vitro LM models severely limits the development of novel therapeutic approaches. To overcome this bottleneck, we employ iteratively selected, clinically relevant in vitro xenograft models that reproduce the features of LM in vivo.^7^ We show that leptomeningeal metastatic cells retain the dynamic in vivo characteristics of LM, particularly the presence of both adherent and floating phenotypes. Similar observations were previously reported for mammary stem and neoplastic cells, lung and ovarian cancer.^10^, ^16^, ^17^ The long‐term in vitro fractionation confirms that heterogenous population of leptomeningeal cancer cells is maintained through plasticity between floating and adherent phenotypes, although the floating phenotype gives rise preferentially to floating cells (Figure 4B). Floating cells in vitro are characterized by elevated TCA cycle and electron transport chain signatures, contain less ATP, and decelerate their growth in both adherent and nonadherent settings. However, when implanted in vivo, and restored into their natural habitat, floating cells promptly colonize subarachnoid space of experimental animals, leading to brisk development of neurologic symptoms and death. This feature is preserved in breast and lung LM models, despite the extensive manipulation during iterative in vivo‐in vitro expansions. Provocatively, this observation in mouse models is validated through a small retrospective clinical series, suggesting that the floating LM phenotype represents the lethal form of the LM cancer cell. This clinical observation merits further validation in a large, multi‐institution cohort.

The mechanism by which these floating cells wreak havoc on the central nervous system while remaining stubbornly resistant to in vitro culture is open for speculation and further study. Of note, the CSF filled with floating cells in many instances reproduces the environment in late‐stage tumors, characterized by hypoxia and limited nutritional resources.^18^, ^19^ Preserved transcriptomic adaptations in metabolic pathways of floating cancer cells may help them to overcome these in vivo constraints but present a disadvantage in regular cell culture. It is unlikely that suspension or spheroid cultures, alone or with the addition of recombinant growth factors, can faithfully recreate the in vivo situation in a culture dish. Development of patient‐derived models may be dependent on in vivo passaging or maintenance in advanced, multicomponent cell culture systems.^20^, ^21^ Our in vitro and in vivo findings show that the anchorage‐independent nature and distinct metabolic requirements of floating cells allow for brisk outgrowth within a nutritionally and spatially constrained CSF. Further studies employing single‐cell and in situ RNA sequencing and metabolomics are necessary to validate these findings in other immunocompetent or humanized experimental models^22^ and human disease.

## CONFLICT OF INTEREST

Adrienne Boire has consulted for Arix Bioscience (2018), is on the Scientific Advisory Board for Evren Scientific (unpaid), and holds patent applications: 62/258044 and 62/052966. Other authors declare no conflict of interest.

## AUTHOR CONTRIBUTIONS

All authors had full access to the data in the study and take responsibility for the integrity of the data and the accuracy of the data analysis. Conceptualization, J.R. and A.B.; Methodology, J.R. and A.P.; Investigation, J.R., Y.C., F.S., U.S., A.P., and T.B; Formal Analysis, J.R.; Writing ‐ Original Draft, J.R. and A.B.; Writing ‐ Review & Editing, J.R. and A.B.; Validation, X.T. and C.D.; Data Curation, Formal Analysis and Visualization, J.R.; Supervision, A.B.; Funding Acquisition, A.B.

## Supporting information


**Figure S1** Cell fractionation method. (A) Overview of cell fractionation method. Cells were exposed to trypsin/EDTA solution for an optimized amount of time and floating fraction was carefully collected. Adherent fraction was collected by further trypsinization. Fractions enriched for floating and adherent cells were then used for downstream applications. Please, refer to the Materials & Method sections for further details. (B) Representative image of floating PC9 LeptoM fraction, 24 hours after the seeding (scale bar = 100 μm). (C‐E) Plots show the post‐fractionation viability of all fractions in Parental and LeptoM PC9 (C), MDA‐231 (D), and HCC1954 cells (E). Results are from at least three independent experiments and data represent mean ± SEM.

**Figure S2** Stability of floating and adherent fraction over the long‐term fractionation.(A‐B) The plots show stability of long‐term fractionated floating and adherent sub‐cultures of PC9 LeptoM cells. Data represent mean ± SEM, two‐way ANOVA. Data are related to Figure 4.

**Figure S3** qPCR validation of RNA‐seq.(A‐D) Plots show relative gene expression for *MT‐ATP6*, *MT‐ND5*, *MT‐CO1* and *MT‐CO3* in trypsinized unfractionated Parental (Par) and Leptomeningeal (LeptoM) cells, and in fractionated floating (FL) and adherent (AD) Leptomeningeal PC9 cells. Results are from at five independent experiments and data represent mean ± SEM, paired *t* test.

**Figure S4** Overview of the in vivo experiments.(A) Scheme shows in vivo experiment overview. in vitro cultured LeptoM cells were fractionated and 2000 cells was injected into the *cisterna magna* of athymic mice. The mixture of floating and adherent fractions in 1:1 ratio was injected as a control.(B‐C) Plots show in vivo growth curves of intracisternally injected, fractionated PC9 LeptoM (B) and MDA‐231 LeptoM cells (C). Data are related to Figure 6.

Table S1
Table S2

## Data Availability

The accession number for RNA‐sequencing data deposited in NCBI Gene Expression Omnibus is GEO:GSE137020. Raw data are available from the corresponding author upon request.
