# Supplementary figures and images for: Leptomeningeal metastatic cells adopt two phenotypic states
Source: Cancer Rep (Hoboken). 2020 Jan 29;5(4):e1236. doi: 10.1002/cnr2.1236 (PMC7772527; doi:10.1002/cnr2.1236)

A

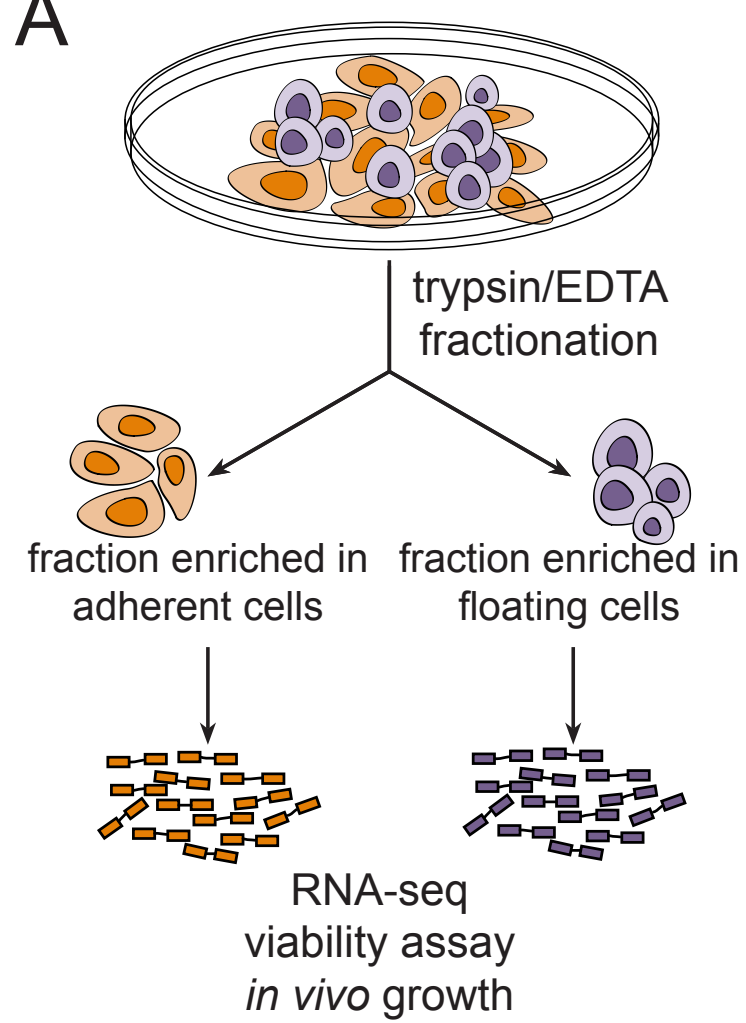

B

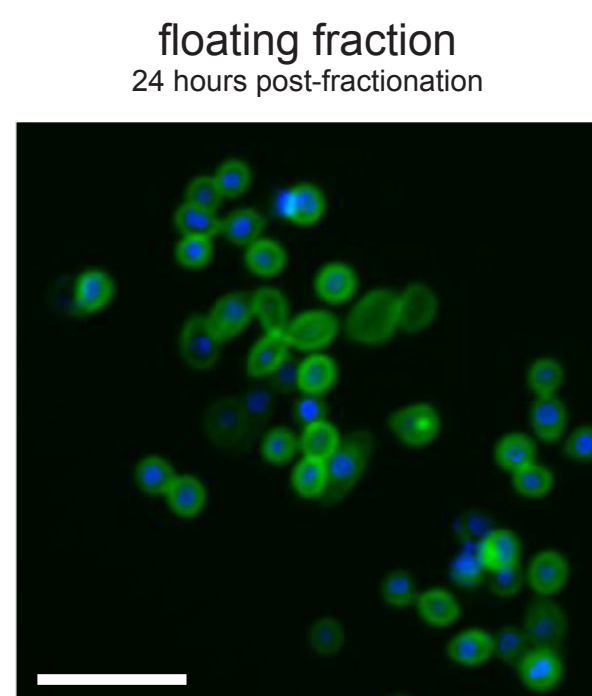

C

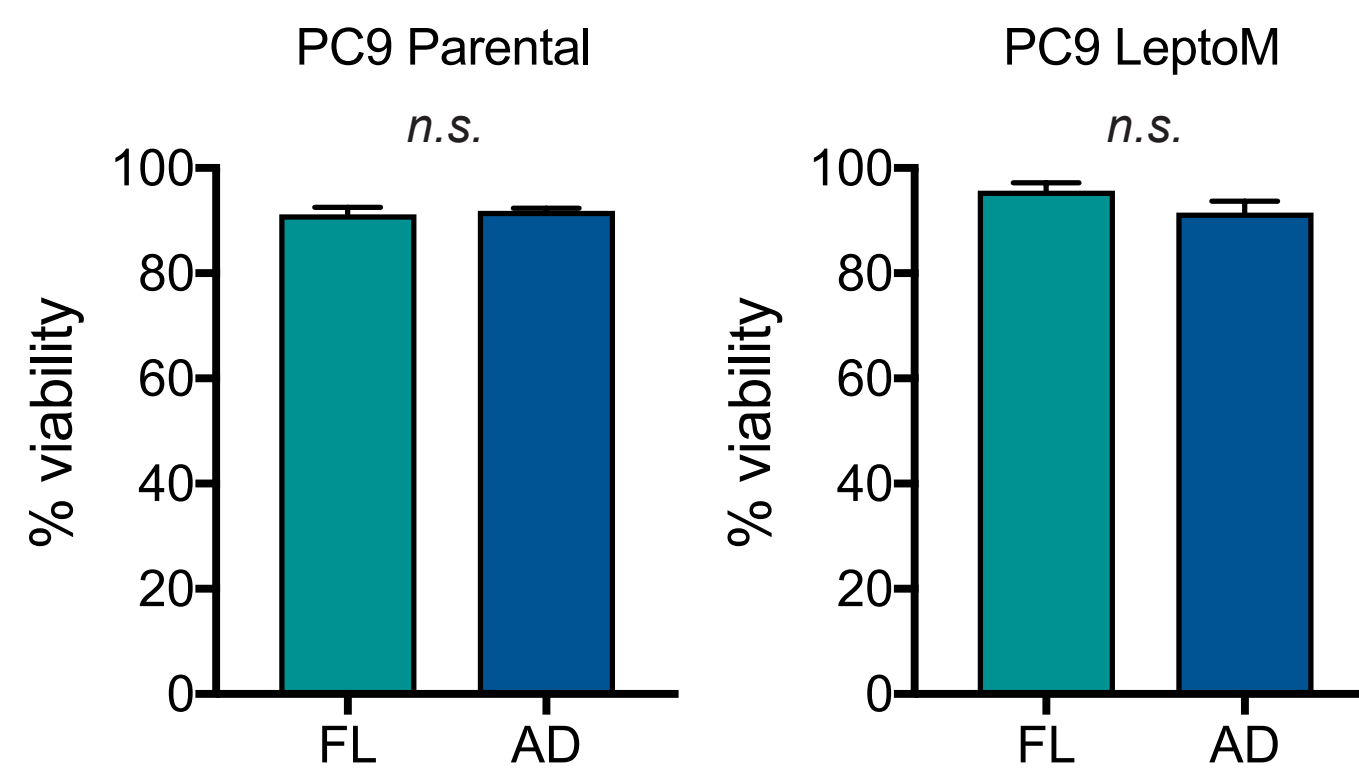

D

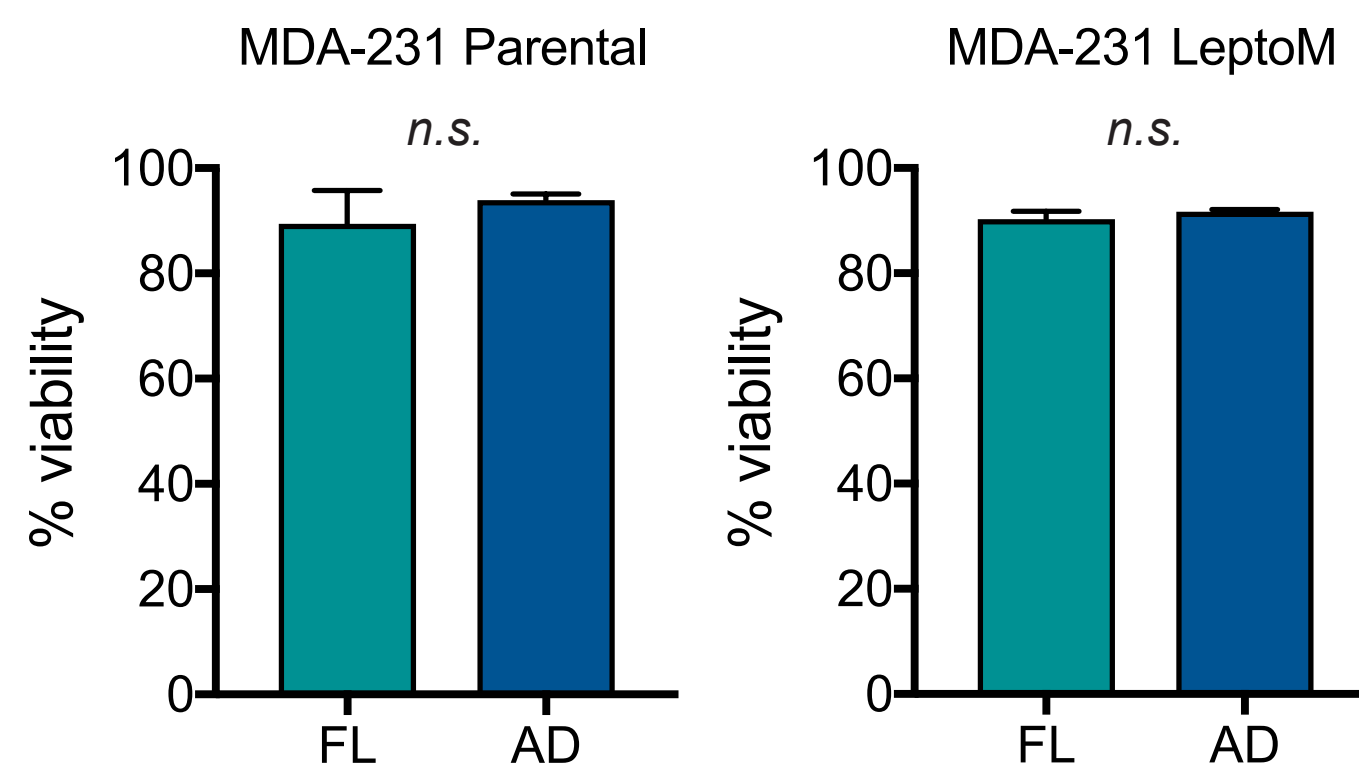

E

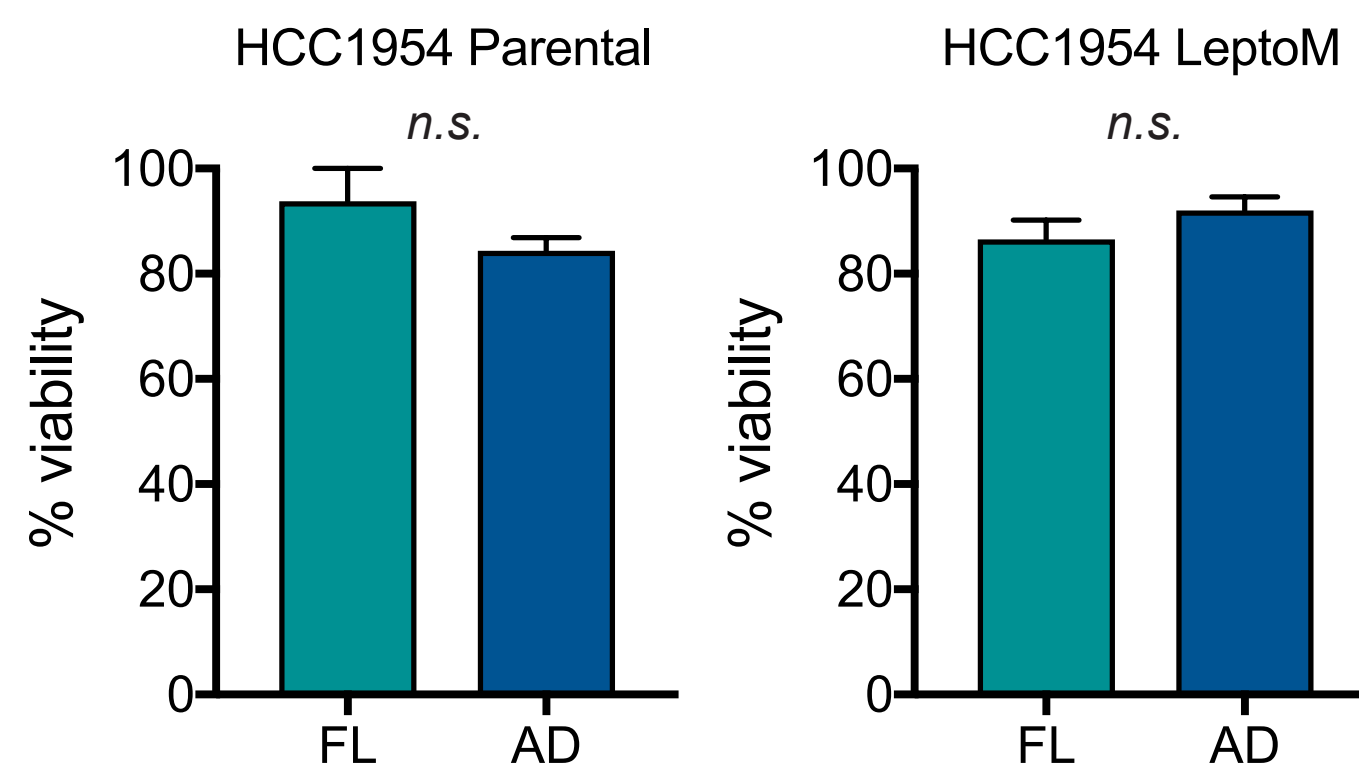

Supplement: Supplementary file 1 — Figure S1 Cell fractionation method. (A) Overview of cell fractionation method. Cells were exposed to trypsin/EDTA solution for an optimized amount of time and floating fraction was carefully collected. Adherent fraction was collected by further trypsinization. Fractions enriched for floating and adherent cells were then used for downstream applications. Please, refer to the Materials & Method sections for further details. (B) Representative image of floating PC9 LeptoM fraction, 24 hours after the seeding (scale bar = 100 μm). (C‐E) Plots show the post‐fractionation viability of all fractions in Parental and LeptoM PC9 (C), MDA‐231 (D), and HCC1954 cells (E). Results are from at least three independent experiments and data represent mean ± SEM. [file CNR2-5-e1236-s003.pdf]

A

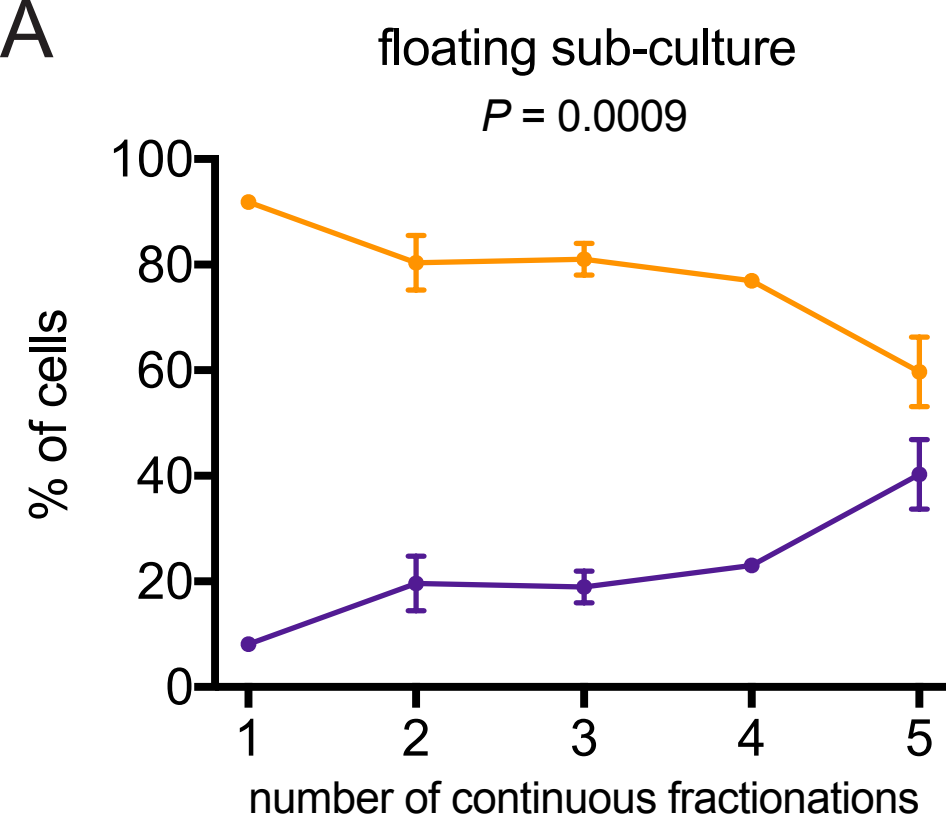

B

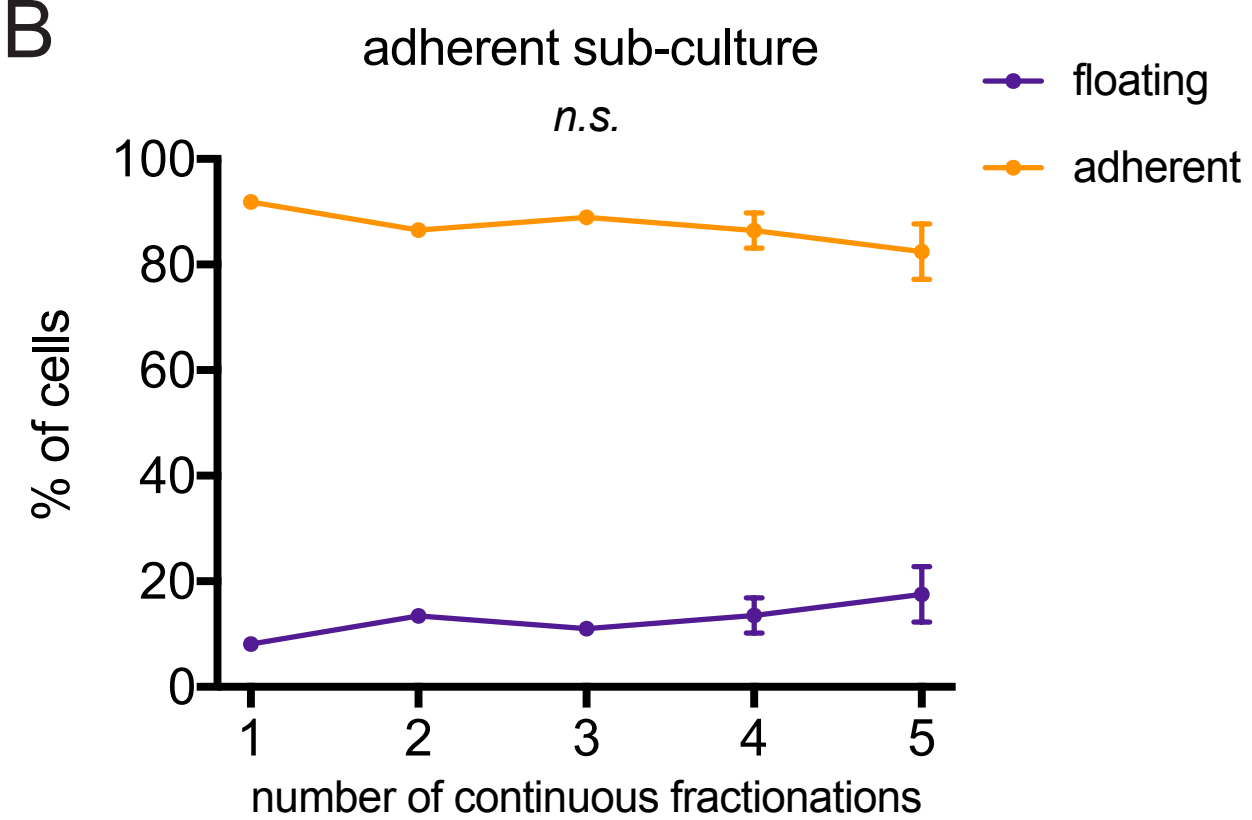

Supplement: Supplementary file 2 — Figure S2 Stability of floating and adherent fraction over the long‐term fractionation.(A‐B) The plots show stability of long‐term fractionated floating and adherent sub‐cultures of PC9 LeptoM cells. Data represent mean ± SEM, two‐way ANOVA. Data are related to Figure 4. [file CNR2-5-e1236-s004.pdf]

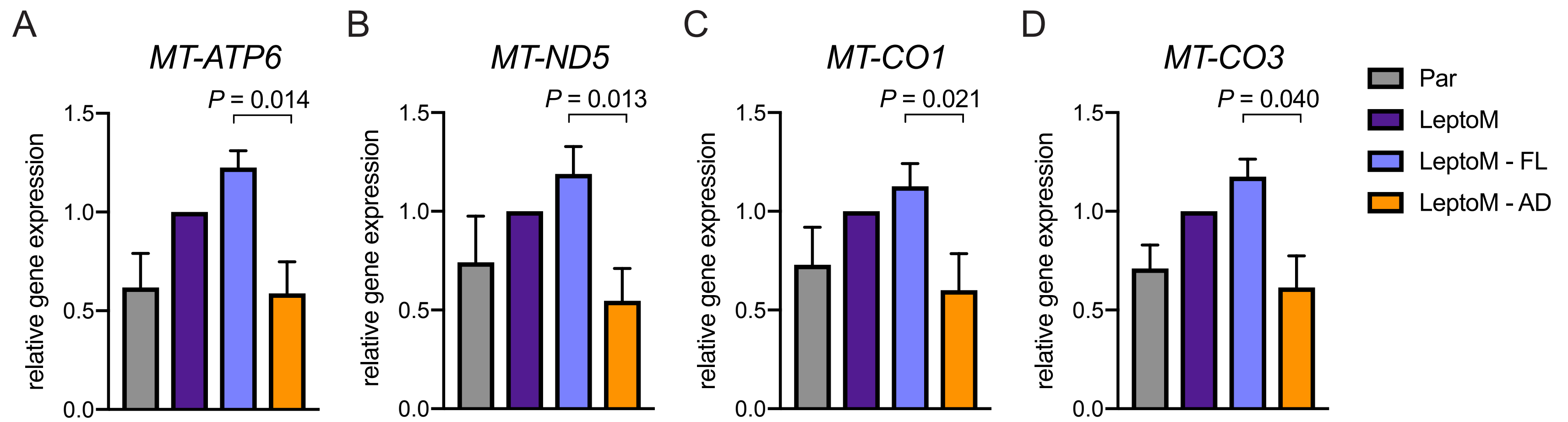

Supplement: Supplementary file 3 — Figure S3 qPCR validation of RNA‐seq.(A‐D) Plots show relative gene expression for MT‐ATP6, MT‐ND5, MT‐CO1 and MT‐CO3 in trypsinized unfractionated Parental (Par) and Leptomeningeal (LeptoM) cells, and in fractionated floating (FL) and adherent (AD) Leptomeningeal PC9 cells. Results are from at five independent experiments and data represent mean ± SEM, paired t test. [file CNR2-5-e1236-s002.pdf]

A

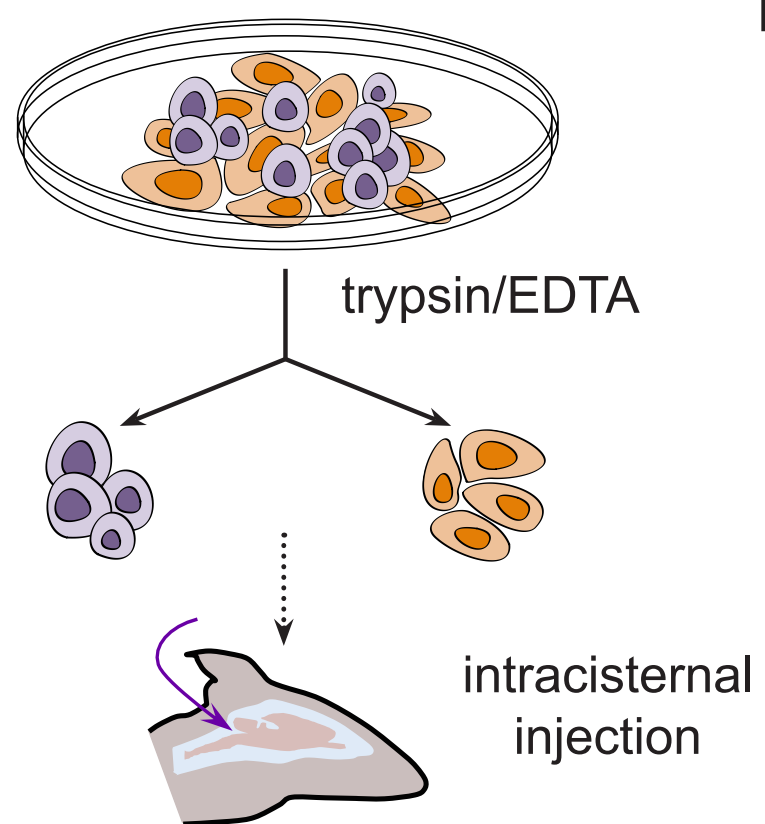

B

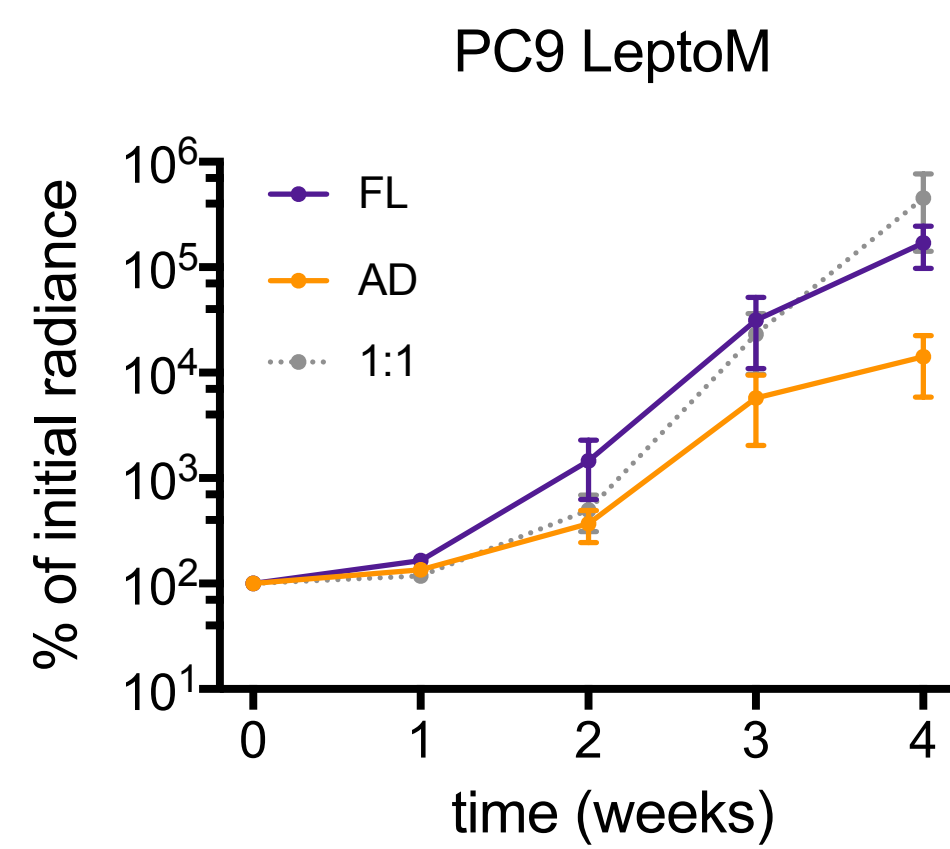

C

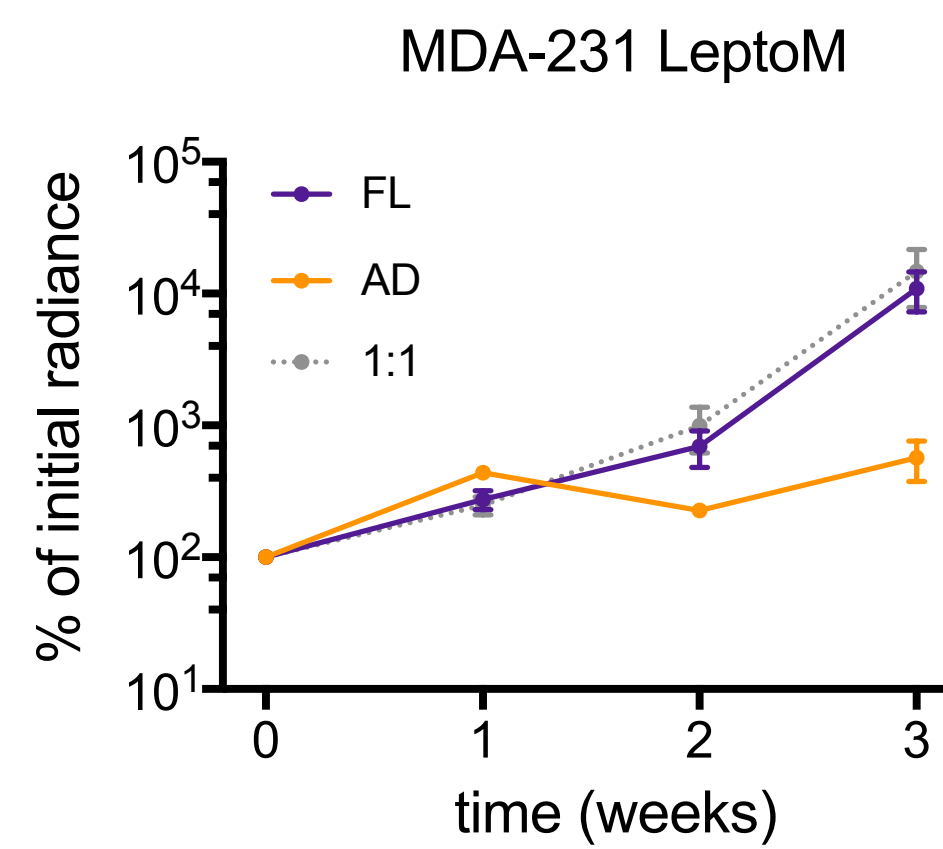

Supplement: Supplementary file 4 — Figure S4 Overview of the in vivo experiments.(A) Scheme shows in vivo experiment overview. in vitro cultured LeptoM cells were fractionated and 2000 cells was injected into the cisterna magna of athymic mice. The mixture of floating and adherent fractions in 1:1 ratio was injected as a control.(B‐C) Plots show in vivo growth curves of intracisternally injected, fractionated PC9 LeptoM (B) and MDA‐231 LeptoM cells (C). Data are related to Figure 6. [file CNR2-5-e1236-s005.pdf]
